# Desigualdades nas Taxas de Mortalidade por Malformações do Sistema Circulatório em Crianças Menores de 20 Anos de Idade entre Macrorregiões Brasileiras

**DOI:** 10.36660/abc.20190351

**Published:** 2020-12-01

**Authors:** Thais Rocha Salim, Thayanne Mendes Andrade, Carlos Henrique Klein, Gláucia Maria Moraes de Oliveira

**Affiliations:** 1 Universidade Federal do Rio de Janeiro Rio de Janeiro Brasil Universidade Federal do Rio de Janeiro, Rio de Janeiro, RJ - Brasil; 2 Escola Nacional de Saúde Pública Rio de JaneiroRJ Brasil Escola Nacional de Saúde Pública, Rio de Janeiro, RJ – Brasil

**Keywords:** Doenças Cardiovasculares, Epidemiologia, Mortalidade Infantil, Crianças, Cardiopatias Congênitas, Serviço de Saúde Pública, Recém-Nascido/tratamento

## Abstract

**Fundamentos:**

Os óbitos por malformações do aparelho circulatório (MAC) em 2015 corresponderam a 43% daqueles por malformações congênitas (MC) em menores de 20 anos de idade no mundo. Os óbitos por MAC apresentam maior impacto sobre a redução da mortalidade, pelo fato de serem evitáveis na maioria das vezes, com o correto diagnóstico e tratamento.

**Objetivo:**

Conhecer a distribuição da mortalidade por MAC por sexo, grupos etários e macrorregiões do Brasil no período de 2000 a 2015, nos menores de 20 anos de idade.

**Métodos:**

Estudo descritivo das taxas de mortalidade por 100 mil e sua mortalidade proporcional, por MAC, outras malformações congênitas (OutMC), doenças do aparelho circulatório (DAC), causas mal definidas (CMD) e causas externas (CE) no Brasil, no período de 2000 a 2015 nos menores de 20 anos. As populações foram obtidas no Instituto Brasileiro de Geografia e Estatística e os óbitos no Departamento de Informática do Sistema Único de Saúde/Ministério da Saúde.

**Resultados:**

Ocorreram 1.367.355 óbitos por todas as causas nos menores de 20 anos de idade, sendo 61,7% do sexo masculino e 55,0% dos óbitos nos menores de 1 ano. Os óbitos por MC em quaisquer órgãos ou sistemas foram 144.057 e os por MAC corresponderam a 39% desses óbitos. Em ambos os sexos, a mortalidade anual por MAC foi de 5,3/100 mil habitantes e a mortalidade proporcional (MP) foi de 4,2%, por DAC 2,2%, por CMD 6,2% e por CE 24,9%. As MAC não especificadas apresentaram as maiores taxas de MP em todas as idades e sexos, notadamente nas regiões Norte e Nordeste (60%). Os óbitos por quaisquer MC ocorreram 5,7 vezes mais no primeiro ano de vida do que nas outras faixas etárias (MAC: 5,0; OutMC: 6,4).

**Conclusão:**

No Brasil, de 2000 a 2015, nos menores de 20 anos de idade, a MAC foi a principal causa de óbito dentre todas as malformações, sendo duas vezes mais importante do que as DAC, principalmente nos menores de 1 ano de idade.A frequência de diagnósticos imprecisos de óbitos por MAC ainda é elevada em todas as idades, sexos, e principalmente nas regiões Norte e Nordeste, o que requer fortalecimento das estratégias de saúde pública e maior atenção ao recém-nascido com objetivo de diagnosticar e instituir tratamento precoce das cardiopatias congênitas com consequente redução na mortalidade. (Arq Bras Cardiol. 2020; 115(6):1164-1173)

## Introdução

No mundo, no ano 2000, foram registrados 10,65 milhões de óbitos por todas as causas nos menores de 20 anos de idade, com redução para 6,65 milhões em 2015, assim que a taxa de mortalidade reduziu de 443,76 por 100 mil habitantes para 269,38 no período.^1^ Essa redução foi atribuída a melhoras no acesso a saúde e educação, redução da pobreza e da taxa de fertilidade.^2 - 4^ Além da redução na taxa de mortalidade global, ocorreram mudanças nas causas dos óbitos que deixaram de ter como etiologia mais frequente as doenças infecciosas e passaram a ter predomínio de causas perinatais como prematuridade e malformações, principalmente nos países com maior desenvolvimento econômico.^2 , 5^

Dentre os óbitos por malformações congênitas, os do aparelho circulatório apresentam maior impacto sobre a possibilidade de redução da mortalidade, por serem evitáveis (com o correto diagnóstico e tratamento) e os mais frequentes.^6 , 7^ Em 2015, as malformações do aparelho circulatório (MAC) corresponderam a 43% dos óbitos por malformações congênitas em menores de 20 anos de idade no mundo. Dentre as crianças que nascem com alguma cardiopatia congênita sem intervenção médica, 14% não sobrevivem ao primeiro mês de vida e 30% ao primeiro ano.^8 , 9^

No Brasil, embora a mortalidade nos menores de 20 anos de idade tenha diminuído nas últimas décadas, a importância relativa das malformações congênitas aumentou, uma vez que, se em 2000 eram a quarta causa de óbito, em 2015 passaram a ser a terceira. Neste último ano, 40% do total destes óbitos foi por MAC.^9 , 10^

As MAC contribuem, com a maior parte, para os óbitos em menores de 1 ano e de 1 a 4 anos, pois essas causas costumam ser incompatíveis com a vida e altamente dependentes de adequado suporte médico hospitalar.^11^ O objetivo deste artigo é conhecer a distribuição por gênero, grupo etário e região, no período de 2000 a 2015, da mortalidade por causas de óbito por MAC em menores de 20 anos de idade. Isso é necessário para instituir medidas de melhoria da assistência em saúde e de redução da mortalidade.

## Material e métodos

Estudo descritivo das taxas de mortalidade e mortalidade proporcional por MAC, outras malformações congênitas (OutMC), doenças do aparelho circulatório (DAC), causas mal definidas (CMD) e causas externas (CE) em menores de 20 anos de idade no Brasil, no período de 2000 a 2015.

As informações referentes aos óbitos foram obtidas no *site* do Departamento de Informática do Sistema Único de Saúde (DATASUS) (http://tabnet.datasus.gov.br/cgi/sim/dados/cid10_indice.htmdados).^12^ Essas informações são compostas pelos conjuntos de todas as declarações de óbito (DO) registradas no Brasil, de 2000 a 2015, ano a ano, em cada macrorregião geoeconômica. Foi utilizada a codificação de causa básica de óbito de acordo com a 10^a^ Revisão da Classificação Estatística Internacional de Doenças e Problemas Relacionados à Saúde da Organização Mundial da Saúde (CID 10).^13^

As informações referentes às populações foram obtidas no *site* (https://www.ibge.gov.br/apps/populacao/projecao);^14^ são projeções realizadas pelo Instituto Brasileiro de Geografia e Estatística com base nos censos e estão disponíveis para o período de 2000 a 2060 por macrorregião brasileira, sexo, faixa etária e pelos totais. Destas, foram utilizadas, de 2000 a 2015, nas faixas etárias 0 a 4 anos, 5 a 9 anos, 10 a 14 anos e 15 a 19 anos em ambos os sexos e em cada macrorregião e no Brasil.^15^ Os limites temporais foram estipulados em função da disponibilidade de informações sobre população, fornecidas pelo IBGE, com metodologia constante, a partir de 2000, e sobre óbitos que obtivemos do DATASUS até 2015.

As informações sobre óbitos foram coletadas em cada macrorregião geoeconômica, em ambos os sexos, e em faixas etárias de menores de 20 anos (menores de 1 ano, de 1 a 4 anos, de 5 a 9 anos, de 10 a 14 anos e de 15 a 19 anos), seguindo a divisão adotada pela Organização Mundial de Saúde (OMS).^15^ Portanto, para o cálculo das taxas de mortalidade na faixa etária de 1 a 4 anos, utilizamos uma aproximação para essa população subtraindo o número de nascidos vivos por sexo e região do total da população de 0 a 4 anos de idade. As taxas de mortalidade nos menores de 1 ano são as mesmas da mortalidade infantil, pois o denominador foi o número de nascidos vivos. A informação sobre os nascidos vivos por sexo e região no período de 2000 a 2015 foi obtida no site do DATASUS.^16^

Os óbitos cuja causa básica correspondem ao capítulo XVII da CID-10 foram divididos em MAC e OutMC.^13^ Os óbitos por MAC foram discriminados nas categorias: câmaras e comunicações cardíacas (Q20); septos cardíacos (Q21); valvas pulmonar e tricúspide (Q22); valvas aórtica e mitral (Q23); outras (Q24 sem 9) e não especificadas (Q24.9); grandes artérias (Q25); e outros vasos (Q26-28). Os óbitos por OutMC compreenderam do Q00-18 e Q30-99. Os óbitos cujas causas básicas foram por DAC correspondem aos do capítulo IX. Os óbitos por CMD correspondem ao capítulo XVIII e os óbitos por CE são aqueles dos capítulos XIX e XX da CID-10.

As mortalidades proporcionais, que correspondem às razões entre óbitos por causas específicas e total de óbitos, foram estimadas de duas formas: as totais (MPt), cujos denominadores incluem todas as causas de óbito, e as endógenas (MPe), cujos denominadores excluem as externas. As taxas de mortalidade por 100 mil (Mort100m) foram estimadas pela razão entre óbitos por causas e populações estimadas. MPt e Mort100mil foram estimados por sexo, faixa etária e macrorregião, correspondentes ao período de 2000 a 2015, enquanto MPe foi obtida ano a ano, por sexo, faixa etária e macrorregião.

Os procedimentos quantitativos foram realizados com os programas Excel-Microsoft^17^ e Stata® versão 14.^18^

O estudo foi feito de acordo com os princípios éticos vigentes e aprovado pelo comitê de Ética em Pesquisa do Hospital Universitário Clementino Fraga Filho pertencente à Universidade Federal do Rio de Janeiro (UFRJ).

## Resultados

No Brasil, de 2000 a 2015, ocorreram 1.367.355 óbitos por todas as causas em menores de 20 anos de idade, sendo 845.481 do sexo masculino e 521.874 do sexo feminino. A importância dos óbitos nos menores de 1 ano de idade caiu de 61,41% no ano 2000 para 51,22% em 2015. A frequência relativa de óbitos foi maior no sexo masculino em todas as faixas etárias, chegando a ser 3,8 vezes maior nos de 15 a 19 anos. A mortalidade anual média por todas as causas foi de 126 por 100 mil habitantes em ambos os sexos; destes óbitos, 61,7% ocorreram no sexo masculino.

No Brasil, os óbitos cuja causa básica foi codificada como malformações congênitas em quaisquer órgãos ou sistemas representaram 144.057 do total. Dos óbitos por quaisquer malformações, 85,8% ocorreram em menores de 1 ano de vida, mantendo distribuição similar entre os sexos. Destes, 57.892 óbitos foram por MAC, correspondendo a 39,0% dos óbitos por malformações. No primeiro ano de vida, os óbitos por quaisquer malformações ocorreram 5,7 vezes mais que nas faixas etárias superiores, sendo que, por MAC, 5,0 vezes, e por OutMC 6,4 vezes. A mortalidade anual por MAC foi de 5,3 óbitos por 100 mil habitantes nos menores de 20 anos de idade em ambos os sexos, de 5,0 no sexo feminino e de 5,6 no masculino. A mortalidade proporcional por MAC, ou seja, o percentual de óbitos por esse grupo de causas em relação ao total de óbitos, foi de 4,2% nos menores de 20 anos de idade, em ambos os sexos, de 5,1 no sexo feminino e de 3,7 no masculino.

Nos menores de 20 anos de idade, as DAC foram a causa de 29.904 óbitos no Brasil; destes, 13.198 no sexo feminino e 16.706 no masculino. A mortalidade proporcional por DAC foi de 2,2% em ambos os sexos, 2,5% no sexo feminino e 2,0% no sexo masculino. O risco de morte por DAC nos menores de um ano foi de 14,7 por 100 mil nascidos vivos, caindo nas faixas etárias seguintes, sendo de 3,9 por 100 mil habitantes na de 15 a 19 anos. Por outro lado, a mortalidade proporcional se elevou de 1,4% nos menores de 1 ano de idade para 3,5% nos de 15 a 19 anos.

As CMD foram dadas como causas em 85.458 óbitos no Brasil, o que correspondeu a 6,2% dos óbitos nos menores de 20 anos de idade; destes, 35.518 no sexo feminino e 49.940 no masculino. O risco de morte por CMD nos menores de 1 ano de idade em ambos os sexos foi de 95,04 por 100 mil nascidos vivos, caindo nas faixas etárias seguintes, sendo de 5,09 por 100 mil habitantes de 15 a 19 anos. Por outro lado, a mortalidade proporcional se elevou de 6% nos menores de 1 ano de idade para 10,8% de 1 a 4 anos com declínio progressivo nas outras faixas etárias.

Ocorreram 340.974 óbitos por CE, sendo 274.627 (80,5%) nos meninos e 66.347 (20%) nas meninas. No Brasil, as CE de óbitos nos menores de 20 anos de idade aumentaram com a progressão da faixa etária em ambos os sexos, porém de forma mais acentuada no sexo masculino. A MP foi 31 vezes maior entre a faixa etária de 15 a 19 anos que nos menores de 1 ano de idade no sexo masculino, enquanto no feminino foi de 18,5 vezes.

Resultados da mortalidade proporcional e das taxas de mortalidade de acordo com causas de óbitos, grupos etários, sexo e regiões do Brasil podem ser vistos nas Tabelas 1 [Table t2] [Table t3] [Table t4] [Table t5] a 6 . As regiões Sul e Centro-Oeste apresentaram quase 2 vezes mais risco de morte por MAC que as regiões Norte e Nordeste nos menores de 1 ano de idade, com redução progressiva desse risco com o aumento da faixa etária.

**Tabela 1:** – Mortalidade proporcional e taxa de mortalidade por grupo de causas em crianças segundo sexo e grupo etário, na região Norte de 2000 a 2015

Causas de óbitos		Masculino					Feminino				
		< 1 ano	1-4 anos	5-9 anos	10-14 anos	15-19 anos	< 1 ano	1-4 anos	5-9 anos	10-14 anos	15-19 anos
MAC	Óbitos MP(%) Mort100mil	2.564 4,9 100,5_(1)_	282 2,7 2,0_(2)_	83 1,5 0,6	55 0,9 0,4	51 0,2 0,4	2.064 5,06 85,3_(1)_	286 3,3 2,1_(2)_	64 1,6 0,5	66 1,6 0,5	39 0,6 0,3
Outras MC	Óbitos MP(%) Mort100mil	4.047 7,7 158,7_(1)_	261 2,5 1,8_(2)_	67 1,2 0,5	50 0,8 0,4	53 0,2 0,4	3.710 9,1 153,4_(1)_	244 2,8 1,8_(2)_	60 1,5 0,4	44 1,1 0,3	26 0,4 0,2
DAC	Óbitos MP(%) Mort100mil	496 0,9 19,4_(1)_	217 2,1 1,5_(2)_	180 3,3 1,2	307 5,0 2,2	635 2,9 4,8	426 1,0 17,6_(1)_	233 2,66 1,69_(2)_	159 4,07 1,15	272 6,54 2,03	457 6,6 3,6
Mal definidas	Óbitos MP(%) Mort100mil	4.548 8,6 178,3_(1)_	1.845 17,6 12,8_(2)_	683 12,5 4,8	722 11,8 5,2	1.475 6,6 11,1	3.452 8,5 142,7_(1)_	1.514 17,3 11,0_(2)_	499 12,8 3,6	493 11,9 3,7	694 10,0 5,4
Externas	Óbitos MP(%) Mort100mil	837 1,6 32,8_(1)_	2.341 22,4 16,3_(2)_	2.133 38,9 14,8	2.794 45,6 20,1	16.668 75,0 125,5	602 1,5 24,9_(1)_	1.402 16,0 10,9_(2)_	1.180 30,2 8,5	1.373 33,0 10,3	2.517 36,4 19,7
Todas as causas	Óbitos MP(%) Mort100mil	52.729 100,0 2067,2_(1)_	10.459 100,0 72,7_(2)_	5.476 100,0 38,1	6.130 100,0 44,1	22.218 100,0 167,3	40.754 100,0 1684,9_(1)_	8.750 100,0 63,5_(2)_	3.911 100,0 28,4	4.158 100,0 31,1	6.914 100,0 54,0

MAC: malformações do aparelho circulatório; OutrasMC: outras malformações congênitas excluindo as MAC; DAC: doenças do aparelho circulatório; MP(%): mortalidade proporcional em percentual;.Mort100mil: taxa de mortalidade por 100 mil (1) Mortalidade por 100 mil nascidos vivos (2) Mortalidade por 100 mil na população de 0 a 4, excluídos os nascidos vivos.

**Tabela 2 t2:** – Mortalidade proporcional e taxas de mortalidade por grupo de causas em crianças segundo sexo e grupo etário, na região Nordeste de 2000 a 2015

Causas de óbitos		Masculino					Feminino				
		< 1 ano	1-4 anos	5-9 anos	10-14 anos	15-19 anos	< 1 ano	1-4 anos	5-9 anos	10-14 anos	15-19 anos
MAC	Óbitos MP(%) Mort100mil	6.743 4,5 93,3_(1)_	822 3,4 2,4_(2)_	255 1,8 0,6	182 1,0 0,4	193 0,2 0,4	5.602 4,9 81,5_(1)_	937 4,6 2,8_(2)_	234 2,3 0,6	180 1,6 0,4	157 0,8 0,4
Outras MC	Óbitos MP(%) Mort100mil	11.086 7,5 153,4_(1)_	758 5,7 2,2_(2)_	256 1,9 0,6	175 1,0 0,4	171 0,2 0,4	9.819 8,6 143,0_(1)_	770 3,7 2,3_(2)_	223 2,2 0,5	172 1,6 0,4	151 0,8 0,4
DAC	Óbitos MP(%) Mort100mil	1.138 0,8 15,7_(1)_	721 3,0 2,1_(2)_	565 4,1 1,3	1.005 5,6 2,3	2374 3,0 5,6	986 0,9 14,4_(1)_	692 3,4 2,1_(2)_	493 5,0 1,2	845 7,7 2,0	1.595 8,0 3,8
Mal definidas	Óbitos MP(%) Mort100mil	12.576 8,5 174,1_(1)_	3.403 14,2 9,9_(2)_	1.378 10,0 3,2	1.429 7,9 3,3	3.357 4,3 7,9	9.545 8,4 139,0_(1)_	2.931 14,3 8,9_(2)_	1.071 10,8 2,6	1.118 10,1 2,7	1.800 9,1 ,3
Externas	Óbitos MP(%) Mort100mil	1.994 1,3 27,6_(1)_	4.895 20,4 14,3_(2)_	5.245 38,1 12,3	9.119 50,5 21,1	62.854 80,3 147,4	1.372 1,2 20,0_(1)_	3.044 14,8 9,2_(2)_	2.627 26,4 6,4	3.380 30,7 8,1	7.621 38,3 18,3
Todas as causas	Óbitos MP(%) Mort100mil	148.346 100 2.053,1_(1)_	23.957 100 70,0_(2)_	13.782 100 32,0	18.038 100 42,0	78.248 100 183,5	113.735 100 1.656,6_(1)_	20.529 100 62,1_(2)_	9.949 100 24,1	11.019 100 26,4	19.872 100 47,8

MAC: malformações do aparelho circulatório; OutrasMC: outras malformações congênitas excluindo as MAC; DAC: doenças do aparelho circulatório; MP(%): mortalidade proporcional em percentual;.Mort100mil: taxa de mortalidade por 100 mil (1) Mortalidade por 100 mil nascidos vivos (2) Mortalidade por 100 mil na população de 0 a 4, excluídos os nascidos vivos.

**Tabela 3 t3:** – Mortalidade proporcional e taxa de mortalidade por grupo de causas em crianças segundo sexo e grupo etário, na região Sudeste de 2000 a 2015

Causas de óbitos		Masculino					Feminino				
		< 1 ano	1-4 anos	5-9 anos	10-14 anos	15-19 anos	< 1 ano	1-4 anos	5-9 anos	10-14 anos	15-19 anos
MAC	Óbitos MP(%) Mort100mil	10.534 7,1 110,0(1)	1.091 4,7 2,7(2)	250 1,8 0,5	250 1,2 0,5	218 0,2 0,4	9.088 7,8 99,5(1)	1.101 5,7 2,8(2)	275 2,6 0,5	218 1,7 0,4	183 0,7 0,3
Outras MC	Óbitos MP(%) Mort100mil	15.407 10,4 160,8(1)	1.252 5,4 3,1(2)	366 2,6 0,7	301 1,5 0,6	290 0,3 0,5	14.042 12,0 153,7(1)	1246 6,4 3,2(2)	372 3,5 0,7	288 2,3 0,5	263 1,1 0,5
DAC	Óbitos MP(%) Mort100mil	1.537 1,0 16,0(1)	818 3,6 2,0(2)	533 3,8 1,0	980 4,8 1,8	2.579 2,6 4,6	1.401 1,2 15,3(1)	784 4,1 2,0(2)	534 5,0 1,1	723 5,7 1,4	1.585 6,5 2,9
Mal definidas	Óbitos MP(%) Mort100mil	6.05 4 4,1 63,2(1)	1.821 7,9 4,5(2)	720 5,1 1,4	1.166 5,7 2,2	3.856 3,9 6,9	4.441 3,8 48,6(1)	1.406 7,3 3,6(2)	650 6,1 1,3	837 6,6 1,6	1.610 6,6 3,0
Externas	Óbitos MP(%) Mort100mil	4.969 3,4 51,9(1)	5.237 22,8 12,8(2)	5.166 36,5 9,8	10.487 51,0 19,4	80.167 80,4 143,6	3.237 2,8 35,4(1)	3.470 17,9 8,9(2)	2.897 27,3 5,7	4.158 32,9 8,0	10.563 43,0 19,5
Todas as causas	Óbitos MP(%) Mort100mil	147.871 100 1.543,8(1)	23.006 100 56,3(2)	14.167 100 26,9	20.571 100 38,0	99.661 100 178,6	116.679 100 1.277,4(1)	19.333 100 49,4(2)	10.663 100 21,1	12.650 100 24,2	24.550 100 45,3

MAC: malformações do aparelho circulatório; OutrasMC: outras malformações congênitas, excluindo as MAC; DAC: doenças do aparelho circulatório; MP(%): mortalidade proporcional em percentual; Mort100mil: taxa de mortalidade por 100 mil (1) Mortalidade por 100 mil nascidos vivos (2) Mortalidade por 100 mil na população de 0 a 4, excluídos os nascidos vivos

**Tabela 4 t4:** – Mortalidade proporcional e taxa de mortalidade por grupo de causas em crianças segundo sexo e grupo etário, na região Sul de 2000 a 2015

Causas de óbitos		Masculino					Feminino				
		< 1 ano	1-4 anos	5-9 anos	10-14 anos	15-19 anos	< 1 ano	1-4 anos	5-9 anos	10-14 anos	15-19 anos
MAC	Óbitos MP(%) Mort100mil	3.869 *8,5* *120,8*_(1)_	429 *5,5* *3,1*_(2)_	114 *2,2* *0,6*	94 *1,2* *0,5*	127 *0,4* *0,6*	3.147 *8,7* *103,1*_(1)_	409 *6,6* *3,1*_(2)_	114 *3,1* *0,7*	80 *1,7* *0,4*	76 *0,9* *0,4*
Outras MC	Óbitos Mort100mil	5.694 *12,5* *177,8*_(1)_	590 *7,6* *4,3*_(2)_	188 *3,7* *1,0*	147 *2,0* *0,8*	141 *0,4* *0,7*	5.093 *14,1* *167,0*_(1)_	495 *8,0* *3,8*_(2)_	158 *4,3* *0,9*	145 *3,1* *0,8*	116 *1,3* *0,6*
DAC	Óbitos MP(%) Mort100mil	257 *0,6* *8,0*_(1)_	191 *2,5* *1,4*_(2)_	123 *2,4* *0,7*	255 *3,4* *1,3*	618 *2,0* *3,2*	233 *0,6* *7,6*_(1)_	184 *2,9* *1,4*_(2)_	118 *3,2* *0,7*	197 *4,3* *1,1*	358 *4,1* *1,9*
Mal definidas	Óbitos MP(%) Mort100mil	1.802 *4,0* *56,3*_(1)_	375 *4,8* *2,7*_(2)_	130 *2,5* *0,7*	210 *2,8* *1,1*	577 *1,8* *3,0*	1.388 *3,9* *45,5*_(1)_	370 *5,9* *2,8*_(2)_	110 *3,0* *0,6*	163 *3,5* *0,9*	293 *3,3* *1,6*
Externas	Óbitos MP(%) Mort100mil	2.037 *4,5* *63,6*_(1)_	2.211 *28,5* *16,0*_(2)_	2.265 *44,3* *12,5*	4.272 *56,8* *22,5*	25.974 *82,3* *133,1*	1.532 *4,3* *50,2*_(1)_	1.332 *21,4* *10,1*_(2)_	1.250 *33,9* *7,2*	1.825 *39,5* *10,0*	4.398 *50,3* *23,4*
Todas as causas	Óbitos MP(%) Mort100mil	45.436 *100,0* *1.419,1*_(1)_	7.749 *100,0* *56,3*_(2)_	5.113 *100,0* *28,2*	7.514 *100,0* *39,6*	31.541 *100,0* *161,6*	36.020 *100,0* *1.180,9*_(1)_	6.228 *100,0* *47,3*_(2)_	3.692 *100,0* *11,2*	4.621 *100,0* *25,4*	8.738 *100,0* *46,5*

MAC: malformações do aparelho circulatório; OutrasMC: outras malformações congênitas, excluindo as MAC; DAC: doenças do aparelho circulatório; MP(%): mortalidade proporcional em percentual; Mort100mil: taxa de mortalidade por 100 mil (1) Mortalidade por 100 mil nascidos vivos (2) Mortalidade por 100 mil na população de 0 a 4, excluídos os nascidos vivos

**Tabela 5 t5:** – Mortalidade proporcional e taxa de mortalidade por grupo de causas em crianças segundo sexo e grupo etário, na região Centro-Oeste de 2000 a 2015

Causas de óbitos		Masculino					Feminino				
		< 1 ano	1-4 anos	5-9 anos	10-14 anos	15-19 anos	< 1 ano	1-4 anos	5-9 anos	10-14 anos	15-19 anos
MAC	Óbitos MP(%) Mort100mil	2.434 8,0 129,8(1)	277 4,9 3,5(2)	64 1,9 0,6	44 1,0 0,4	52 0,3 0,5	2.115 8,8 118,6(1)	239 5,2 3,2(2)	41 1,8 0,4	49 1,7 0,5	51 1,0 0,5
Outras MC	Óbitos MP(%) Mort100mil	3.481 11,4 185,7(1)	272 4,8 3,5(2)	79 2,3 0,8	52 1,1 0,5	53 0,3 0,5	3.065 12,7 171,9(1)	254 5,5 3,4(2)	57 2,5 0,6	71 2,5 0,7	44 0,8 0,4
DAC	Óbitos MP(%) Mort100mil	307 1,0 16,4(1)	137 2,4 1,7(2)	92 2,7 0,9	202 4,5 2,0	439 2,3 4,2	234 1,0 13,1(1)	152 3,3 2,0(2)	72 3,1 0,7	160 5,6 1,6	305 5,8 3,0
Mal definidas	Óbitos MP(%) Mort100mil	927 3,0 49,4(1)	248 4,4 3,2(2)	108 3,2 1,1	152 3,4 1,5	378 1,9 3,7	588 2,4 33,0(1)	210 4,6 2,8(2)	78 3,4 0,8	89 3,1 0,9	168 3,2 1,7
Externas	Óbitos MP(%) Mort100mil	979 3,2 52,2(1)	1.620 28,5 20,7(2)	1.575 46,8 15,8	2.615 57,8 25,9	16.173 83,6 156,5	688 2,9 38,6(1)	1.080 23,5 14,4(2)	847 36,8 8,9	1.256 44,1 12,9	2.696 51,0 26,7
Todas as causas	Óbitos MP(%) Mort100mil	30.511 100,0 1.627,5(1)	5.683 100,0 72,5(2)	3.366 100,0 33,8	4.522 100,0 44,8	19.348 100,0 187,2	24.081 100,0 1.350,7(1)	4.590 100,0 61,2(2)	2.303 100,0 24,1	2.845 100,0 29,2	5.290 100,0 52,4

MAC: malformações do aparelho circulatório; OutrasMC: outras malformações congênitas excluindo as MAC; DAC: doenças do aparelho circulatório; MP(%): mortalidade proporcional em percentual; Mort100mil: taxa de mortalidade por 100 mil (1) Mortalidade por 100 mil nascidos vivos (2) Mortalidade por 100 mil na população de 0 a 4, excluídos os nascidos vivos

**Tabela 6:** – Mortalidade proporcional e taxa de mortalidade por grupo de causas em crianças segundo sexo e grupo etário, Brasil de 2000 a 2015

Causas de óbitos		< 20 anos	Masculino						Feminino					
		Total	Total	< 1 ano	1-4 anos	5-9 anos	10-14 anos	15-19 anos	Total	< 1 ano	1-4 anos	5-9 anos	10-14 anos	15-19 anos
MAC	Óbitos MP(%) Mort100mil	57.892 4,2 5,3	31.077 3,7 5,62	26.144 6,2 107,0(1)	2.901 4,1 2,7(2)	766 1,8 0,6	625 1,1 0,4	641 0,3 0,5	26.815 5,15 5,0	22.016 6,6 94,7(1)	2.972 5,0 2,9(2)	728 2,4 0,5	593 1,7 0,4	506 0,8 0,4
Outras MC	Óbitos MP(%) Mort100mil	86.165 6,3 7,9	45.237 5,4 8,2	39.715 9,3 162,6(1)	3.133 4,4 2,9(2)	956 2,3 0,7	725 1,3 0,5	708 0,3 0,5	40.928 7,78 7,7	35.729 10,8 153,7(1)	3.009 5,1 2,9(2)	870 2,9 0,7	720 2,0 0,5	600 0,9 0,4
DAC	Óbitos MP(%) Mort100mil	29.904 2,2 2,8	16.706 2,0 3,0	3.735 0,9 15,3(1)	2.084 2,9 1,9(2)	1.493 3,6 1,1	2.749 4,8 2,0	6.645 2,6 4,7	13.198 2,54 2,5	3.280 1,0 14,1(1)	2.045 3,4 2,0(2)	1.376 4,5 1,0	2.197 6,2 1,6	4.300 6,6 3,1
Mal definidas	Óbitos MP(%) Mort100mil	85.458 6,2 7,9	49.940 5,9 9,0	25.907 6,1 106,0(1)	7.692 10,9 7,0(2)	3.019 7,2 2,2	3.679 6,5 2,6	9.643 3,8 6,8	35.518 6,82 6,7	19.414 5,9 83,5(1)	6.431 10,8 6,2(2)	2.408 7,9 1,8	2.700 7,7 2,0	4.565 7,0 3,3
Externas	Óbitos MP(%) Mort100mil	340.974 24,9 31,4	274.627 32,5 49,7	10.816 2,5 44,3 (1)	16.304 23,0 15,0(2)	16.384 39,1 11,9	29.287 51,6 20,9	201.836 80,4 142,5	66.347 12,75 12,5	7.431 2,2 32,0(1)	10.328 17,4 9,9(2)	8.801 28,8 6,6	11.992 34,0 8,9	27.795 42,5 20,2
Todas as causas	Óbitos MP(%) Mort100mil	1.367.355 100,0 126,0	845.481 100,0 153,0	424.932 100,0 1.739,3(1)	70.854 100,0 65,2(2)	41.904 100,0 30,4	56.775 100,0 40,5	251.016 100,0 177,3	521.874 100,0 98,0	331.269 100,0 1424,7(1)	59.430 100,0 57,0(2)	30.518 100,0 23,0	35.293 100,0 26,1	65.364 100,0 47,6

MAC: malformações do aparelho circulatório; OutrasMC: outras malformações congênitas excluindo as MAC; DAC: doenças do aparelho circulatório; MP(%): mortalidade proporcional em percentual; Mort100mil: taxa de mortalidade por 100 mil (1) Mortalidade por 100 mil nascidos vivos (2) Mortalidade por 100 mil na população de 0 a 4, excluídos os nascidos vivos

**Figura 1:**
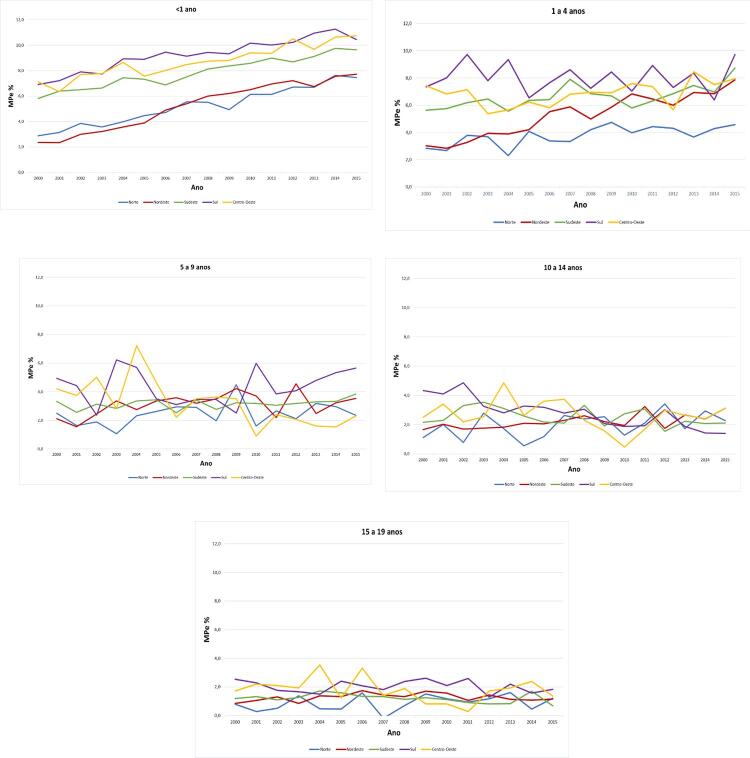
– Mortalidade proporcional endógena por malformações do aparelho circulatório por faixa etária e macrorregião do Brasil de 2000 a 2015. MPe=mortalidade proporcional endógena, mortalidade proporcional, excluídas as causas externas

A variação ao longo do tempo da MPe por MAC, excluídas as causas externas, mostrou aumento de 1,5 vez nos menores de 1 ano de idade em ambos os sexos nas regiões Sul, Centro-Oeste e Sudeste do ano 2000 para 2015. Na região Norte, esse aumento foi de 2,6 vezes e, na região Nordeste, de 3,2 vezes. Como não foi encontrada diferença relevante entre os sexos, optou-se por apresentar o resultado independente do sexo. Nas demais faixas etárias, as variações ao longo do tempo foram discretas, com alguns picos isolados, devido às baixas frequências de óbito. Nos menores de 1 ano de idade, a diferença entre as MPe das regiões Sul e Nordeste era de 4,6% no ano 2000 e caiu para 2,8% em 2015, assim como nos de 15 a 19 anos caiu de 1,7% para 0,6% de 2000 para 2015 ( Figura 1 ). De forma geral as diferenças das MPe entre as regiões diminuíram em todas as faixas etárias especialmente nos últimos anos observados.

**Figura 2:**
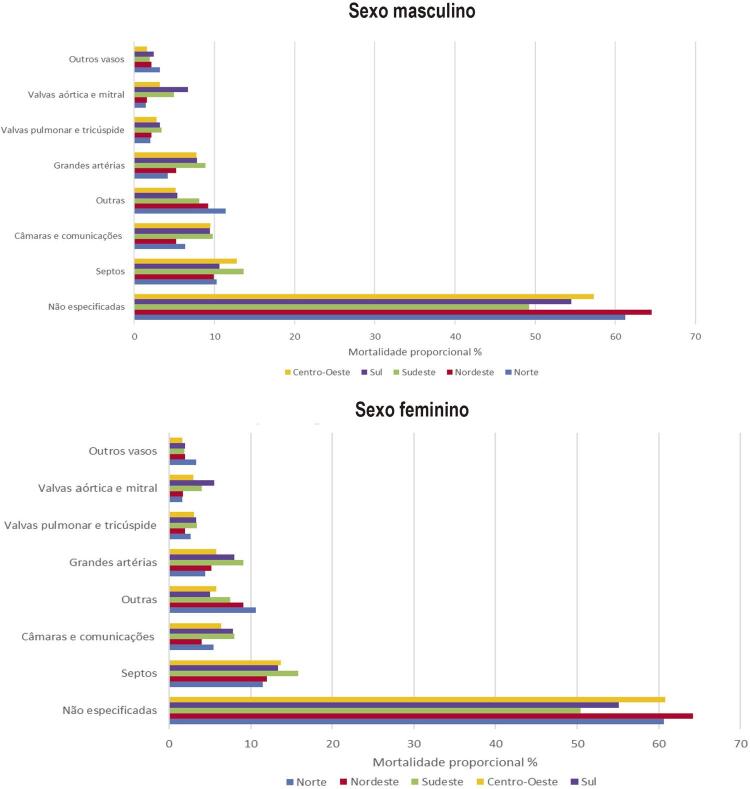
– Mortalidade proporcional anual decorrente de causas específicas de malformações do sistema circulatório em menos de 20 anos de idade por macrorregião do Brasil, 2000 a 2015.

Entre as MAC, a maior mortalidade proporcional ocorreu sem diagnóstico preciso, denominado não especificado de acordo com a CID 10, em todas as regiões, independentemente de sexo e idade. No Norte e no Nordeste, mais de 60% dos óbitos por MAC não foram classificados de forma específica (Q24.9). A segunda categoria de MAC mais frequente foi a malformação de septos cardíacos em todas as regiões, independentemente de sexo e idade, de forma mais acentuada na região Sudeste com frequência de 13% ( Figura 2 ).

## Discussão

No Brasil, de 2000 a 2015, os óbitos em menores de 20 anos de idade se concentraram em mais da metade dos casos, 55%, nos menores de 1 ano de idade, demonstrando o quanto essa faixa etária é vulnerável. Observou-se um padrão de distribuição semelhante em relação ao grupo etário e sexo da mortalidade por MAC nas distintas regiões do Brasil. O mesmo se aplica às demais causas de óbito. Entretanto, as evoluções das importâncias relativas dos grandes grupos de causas de óbito variaram de forma diferente com a progressão da idade, independentemente do sexo. Assim, as malformações, MAC e outras MC, diminuíram de importância com o aumento da idade nos menores de 20 anos de idade. Já com as DAC ocorreu o contrário, enquanto as causas externas descreveram um J, e as CMD pouco variaram com o máximo no grupo de 1 a 4 anos.

Apesar de os riscos de óbito por todas as causas terem sido maiores nos indivíduos do sexo masculino, a importância relativa dos óbitos por malformações e doenças do aparelho circulatório foram maiores nos indivíduos do sexo feminino devido ao predomínio das causas externas entre aqueles do sexo masculino, de forma crescente com o aumento da idade. Essa observação coincide com o verificado em outro estudo.^19^ O predomínio do sexo masculino na mortalidade por CE tem sido observado em diversas partes do mundo.^1 , 4 , 10 , 20^ Tal fato pode ser atribuído a maior exposição desse sexo a fatores de risco, como acidentes, consumo de álcool, fumo ou outras drogas, uso de armas de fogo ou brancas, evasão escolar e inserção em atividades consideradas ilícitas.^21^

A distribuição da mortalidade proporcional por MAC nas regiões do Brasil correspondeu ao encontrado na América Latina. Segundo dados fornecidos pelo *Global Burden of Disease (GBD)* , em 2015, em menores de 20 anos de idade, o México apresentou mortalidade proporcional de 9,7% por MAC, seguido pelo Sul da América Latina (Argentina, Chile e Uruguai) com 7,8%, Brasil com 6,5%, América Andina (Bolívia, Equador e Peru) com 5,8% e Caribe com 4,4%.^1^ Portanto, regiões com maiores índices de pobreza apresentaram menor percentual de óbitos por MAC, o que pode ser atribuído a menor capacidade diagnóstica, visto que, para o diagnóstico de MAC, é necessário adequado suporte médico e hospitalar.^5 , 6^ Observe que há diferença entre os percentuais no presente estudo com aqueles do GBD, porque, neste último, foram compilados dados completos de apenas 8 unidades federativas do Brasil, sendo estimados os das demais.^3^

Pela evolução temporal da mortalidade proporcional endógena, parece que a correção do baixo percentual de diagnóstico de óbitos por MAC ocorreu em todas as regiões, com mais intensidade nas regiões Norte e Nordeste, especialmente nos menores de 1 ano e idade – a faixa etária preferencial do óbito por MAC. Entretanto, os percentuais de diagnósticos anatômicos e funcionais imprecisos de MAC, classificados como não especificados, ainda são mais elevados naquelas regiões. Acresce-se ainda a este fato que os maiores percentuais de óbitos por CMD também se apresentaram em níveis mais elevados nas regiões Norte e Nordeste, nos menores de 5 anos de idade. Portanto, há necessidade de aperfeiçoar os métodos diagnósticos especialmente nas regiões mais pobres do país.

Dentre as causas de óbitos por MAC, as não especificadas são as mais frequentes em todas as idades, sexos e regiões, o que sugere o baixo acesso ao diagnóstico pré-natal ou ao nascimento. De acordo com a Sociedade Brasileira de Pediatria, cerca de 1 a 2 de cada mil nascidos vivos apresentam cardiopatia congênita crítica, e 30% destes recebem alta hospitalar sem diagnóstico da cardiopatia, podendo evoluir para choque, hipóxia ou óbito precoce antes de receber tratamento adequado.^22^ Medidas como a realização do pré-natal e das ecocardiografias obstétricas poderiam reduzir esses óbitos, possibilitando o diagnóstico precoce e a referência dos pacientes para centros especializados de tratamento, mesmo antes do nascimento.^23^

A redução em algumas das diferenças regionais, apontadas nas MPe nos últimos anos, pode ser atribuída a medidas de saúde pública para detecção da cardiopatia congênita, tais como o “teste do coraçãozinho”,^23^ que consiste na aferição da oximetria de pulso de forma rotineira em recém-nascidos com mais de 34 semanas de gestação, recomendada desde 2011 e incorporada à tabela de procedimentos do SUS em 2014.^24^ Outra medida é a realização rotineira de ecocardiografia fetal em gestantes com mais de 35 anos de idade ou outros fatores de risco para malformações fetais.^25^ Em 2004, foi firmado o “Pacto pela Redução da Mortalidade Materna e Neonatal” entre os três níveis de atenção federativos do Brasil, com a meta de reduzir a mortalidade neonatal. As estratégias de obtenção desta meta foram traçadas para reduzir a mortalidade com mais ênfase nas regiões Norte e Nordeste.^26^

As DAC apresentaram comportamento inverso ao das MAC, aumentando em importância de causa de óbito com a progressão da idade. Deve-se considerar que crianças que apresentam MAC, até mesmo já corrigidas, e que não morreram no primeiro ano de vida podem apresentar complicações e sequelas, como insuficiência cardíaca, arritmias, endocardite, entre outras DAC, que podem levar ao óbito na adolescência, aumentando a mortalidade por DAC.^27^ As menores diferenças entre a razão MAC/DAC estão na região Nordeste, seguida da Norte, o que pode ser explicado pela confusão entre os diagnósticos de MAC com o de DAC, provavelmente mais frequente naquelas regiões, uma vez que o diagnóstico diferencial é mais difícil entre estas causas quando os recursos diagnósticos são escassos ou tardios.

Uma limitação deste estudo foi a divisão da faixa etária utilizada para o cálculo das projeções das populações fornecidas pelo IBGE que incluiu os menores de 1 ano de idade no mesmo grupo de menores de 5 anos, o que nos levou a uma aproximação dos valores daqueles de 1 a 4 anos, para valores ligeiramente menores do que os reais. Desta forma, os riscos medidos pelas taxas de mortalidade por 100 mil nesta faixa etária foram ligeiramente superestimados. Entretanto, esse problema não afetou as estimações de mortalidades proporcionais, pois estas não dependem de estimativas de população. Outra limitação é a qualidade na informação sobre causas de óbito no preenchimento das DO. O campo do tempo entre diagnóstico e óbito mostrou preenchimento incompleto e não foi possível determinar a influência dessa variável nos diagnósticos imprecisos de MAC na morte. A DO é, no entanto, a única fonte abrangente de dados sobre mortes para o Brasil como um todo. Entretanto, as DO são as únicas fontes abrangentes de dados sobre óbitos disponíveis para o conjunto do Brasil.

## Conclusão

No Brasil, de 2000 a 2015, nos menores de 20 anos de idade, a MAC foi a principal causa de óbito dentre todas as malformações, sendo duas vezes mais importante do que as DAC, principalmente nos menores de 1 ano de idade. Houve melhoras no diagnóstico dos óbitos por MAC nos últimos anos da série. Porém, neste grupo de causas de óbito, a frequência de diagnósticos imprecisos ainda é elevada em todas as idades, sexos e, principalmente, nas regiões Norte e Nordeste. O que requer fortalecimento das estratégias de saúde pública e maior atenção ao recém-nascido, com objetivo de diagnosticar e instituir tratamento precoce das cardiopatias congênitas com consequente redução na mortalidade.

## References

[1] 1. Global Burden of Disease Collaborative Network. Global Burden of Disease Study 2016 (GBD 2016) Results. Seattle, United States: Institute for Health Metrics and Evaluation (IHME). [Cited in 2018 set 10]. Available from http://ghdx.healthdata.org/gbd-results-tool

[2] 2. Marinho F, Passos VMA, Malta DC, França EB, Abreu DMX, et al. Burden of disease in Brazil, 1990-2016: a systematic subnational analysis for the Global Burden of Disease Study 2016. Lancet. 2018; 10.1016/S0140-6736(18)31221-2 PMC6123514 30037735

[3] 3. Kassebaum N, Kyu HH, Zoeckler L, Olsen HE, Thomas K, et al. Child and Adolescent Health From 1990 to 2015: Findings from the Global Burden of Diseases, Injuries, and Risk Factors 2015 Study. JAMA Pediatr. 2017. 1;171(6):573-592. 10.1001/jamapediatrics.2017.0250 PMC5540012 28384795

[4] 4. Tassinari S, Martínez-Vernaza S, Erazo-Morera N, Pinzón-Arciniegas MC, Gracia., Epidemiología de las cardiopatías congénitas en Bogotá, Colombia en el período comprendido entre 2001 y 2014: ¿Mejoría en la vigilancia o aumento en la prevalencia? Biomédica. 2018;38(2):141-8.

[5] 5. Malta DC, Duarte EC, Almeida MF, Dias MAS, Morais Neto OL, Moura L, et al. Lista de causas de mortes evitáveis por intervenções do Sistema Único de Saúde do Brasil. Epidemiol Serv Saúde. 2007; 16:233-4.

[6] 6. Brasil. Ministério da Saúde. Secretaria de Vigilância em Saúde, Secretaria de Atenção à Saúde, Ministério da Saúde. Manual de vigilância do óbito infantil e fetal e do Comitê de Prevenção do Óbito Infantil e Fetal. 2ª ed. Brasília;2009.

[7] 7. Brum Cda A, Stein AT, Pellanda LC. Infant mortality in Novo Hamburgo: associated factors and cardiovascular causes. Arq Bras Cardiol. 2015; 104(4):257-65. 10.5935/abc.20140203 PMC4415861 25993588

[8] 8. Lozano R, Naghavi M, Foreman K, Lim S, Shibuya K, Aboyans V, et al. Global and regional mortality from 235 causes of death for 20 age groups in 1990 and 2010: a systematic analysis for the Global Burden of Disease Study 2010. Lancet.2012;380:2095-128. 10.1016/S0140673612617280. PMC10790329 23245604

[9] 9. Salim TR, Soares GP, Klein CH, Oliveira GMM. Mortalidade por Doenças e Malformações do Aparelho Circulatório em Crianças no Estado do Rio de Janeiro. Arq Bras Cardiol 2016; 106(6):464-73. 10.5935/abc.20160069 PMC4940145 27192384

[10] 10. Brasil.Ministério da Saúde. Datasus: informações de saúde, morbidade e informações epidemiológicas. [Citado em 2018 agosto]. Disponível em: htpp://www.datasus.gov.br.

[11] 11. Organização Mundial de Saúde. (OMS). Classificação estatística internacional de doenças e problemas relacionados à saúde: Classificação Internacional de Doenças. (CID). 10a revisão. São Paulo: EDUSP; 1995.

[12] 12. Instituto Brasileiro de Geografia e Estatística. (IBGE). Projeções populacionais Brasil de 2000-2060. [Citado em 2018 fevereiro]. Disponível em: https://www.ibge.gov.br/apps/populacao/projecao.

[13] 13. World Health Organization.(WHO). Young People´s Health – a Challenge for Society. Report of a WHO Study Group on Young People and Health for All. Geneva;1986. (Technical Report Series 731) 3085358

[14] 14. Brasil. Ministério da Saúde. Datasus. Portal de saúde. Sistema de informações de nascidos vivos. [on line]. [Citado em 2018 fevereiro]. Disponível em: http://www.datasus.gov.br.

[15] 15. Microsoft Corporation Microsoft Excel. Version 2016. Redmond: Washington, 2016.

[16] 16. Statistics/Data Analysis. STATA Corporation: STATA, Version 14. Texas: University of Texas (USA); 2013.

[17] 17. Matos KF, Martins CB. Epidemiological profile of mortality by external causes in children, teenagers and young people in the capital of the State of Mato Grosso, Brazil, 2009. Epidemiol Serv Saúde. 2012;21(1):43-53.

[18] 18. World Health Organization. (WHO). Media Centre. The top 10 causes of death. [on line]. [Cited in 2018 september]. Available from: http://www.who. int/mediacentre.

[19] 19. Van Hedel K, van Lenthe FJ, Groeniger JO, Mackenbach JP. What’s the difference? A gender perspective on understanding educational inequalities in all-cause and cause-specific mortality. BMC Public Health. 2018; 18:1105. 10.1186/s12889-018-5940-5. PMC6131918 30200912

[20] 20. Diagnóstico precoce de cardiopatia congênita crítica: oximetria de pulso como ferramenta de triagem neonatal. Departamentos de Cardiologia e Neonatologia da SBP.[Citado em 23 set 2018]. Disponível em: http://www.sbp.com.br/pdfs/diagnostico-precoce-oximetria.pdf.

[21] 21. Victora CG, Aquino EML, Leal MC, Monteiro CA, Barros FC, et al. Saúde de mães e crianças no Brasil: progressos e desafios. Lancet. May 2011;32-46. DOI:10.1016/S01406736(11)60138-4

[22] 22. Reller MD, Strickland JM, Riehle-Colarusso T, Mahle WT, Correa A. Prevalence of Congenital Heart Defects in Metropolitan Atlanta, 1998–2005. Pediatr. 2008; 153(6): 807–13. 10.1016/j.jpeds.2008.05.059 PMC2613036 18657826

[23] 23. Departments of Cardiology and Neonatology of SBP. Early diagnosis of critical congenital heart disease: pulse oximetry as a neonatal screening tool. [online]. [Cited in september 2018]. Available at: http://www.sbp.com.br/pdfs/diagnostico-precoce-oximetria.pdf.

[24] 24. Brasil. Ministério da Saúde Departamento de Gestão e Incorporação de Tecnologias em Saúde da Secretaria de Ciência, Tecnologia e Insumos Estratégicos – DGITS/SCTIE Comissão Nacional de Incorporação de Tecnologias no SUS (CONITEC) - Relatório n° 115.

[25] 25. Camarozano A, Rabischoffsky A, Maciel BC, Brindeiro Filho D, Horowitz ES, Pena JLB, et al. Sociedade Brasileira de Cardiologia. Diretrizes das indicações da ecocardiografia. Arq Bras Cardiol.2009;93(6 supl.3):e265-e302.

[26] 26. Brasil.Ministério da saúde secretaria de atenção à saúde departamento de ações programáticas estratégicas. Pacto pela Redução da Mortalidade Materna e Neonatal. Brasília, 2004.

[27] 27. Vetter VL, Covington TM, Dugan NP, Haley DM, Dykstra H, Overpeck M, et al. Cardiovascular deaths in children: general overview from the National Center for the Review and Prevention of Child Deaths. Am Heart J. 2015;169(3):426-37. 10.1016/j.ahj.2014.11.014 25728734

