# Supplementary material for: Desigualdades nas Taxas de Mortalidade por Malformações do Sistema Circulatório em Crianças Menores de 20 Anos de Idade entre Macrorregiões Brasileiras
Source: Arq Bras Cardiol. 2020 Dec 1;115(6):1164–73. [Article in Portuguese] doi: 10.36660/abc.20190351 (PMC8133742; doi:10.36660/abc.20190351)
Supplement: Translation

# Translation: Inequalities in Mortality Rates from Malformations of Circulatory System Between Brazilian Macroregions in Individuals Younger Than 20 Years

**Authors:** Thais Rocha Salim, Thayanne Mendes Andrade, Carlos Henrique Klein, Gláucia Maria Moraes de Oliveira

**Affiliations:** 1 Brazil Universidade Federal do Rio de Janeiro, Rio de Janeiro, RJ - Brazil; 2 Brazil Escola Nacional de Saúde Pública, Rio de Janeiro, RJ – Brazil

**Keywords:** Cardiovascular Diseases, Epidemiology, Infant Mortality, Children, Heart Defects Congenital, Public Health Service, Infant Newborn/treatment

## Abstract

**Background:**

Deaths from malformations of the circulatory system (MCS) have a major impact on mortality reduction. given that most cases are avoidable with correct diagnosis and treatment.

**Objectives:**

To describe the distribution of mortality from MCS by sex. age. and macroregion in Brazil. in individuals under the age of 20. between 2000 and 2015.

**Methods:**

A descriptive study of mortality rates and proportional mortality (PM) from MCS. other congenital malformations (OCM). circulatory system disease (CSD). ill-defined causes (IDC). and external causes (EC) in Brazil.

**Results:**

There were 1.367.355 deaths from all causes in individuals younger than 20. 55.0% under 1 year of age. A total of 144.057 deaths were caused by congenital malformations. 39% of them by MCS. In both sexes. the annual mortality from MCS was 5.3/100.000. PM from MCS was 4.2%. CSD 2.2%. IDC 6.2% and EC 24.9%. Unspecified MCS showed the highest PM rates in both sexes and age groups. especially in the north and northeast regions (60%). Deaths from malformations occurred 5.7 times more frequently during the first year of life than in other ages (MCS: 5.0; OCM: 6.4).

**Conclusions:**

MCS was the leading cause of death among all malformations. being twice as important as CSD. mainly under 1 year of age. The frequency of misdiagnosis of MCS as cause of death was high in all ages and both sexes. especially in the north and northeast regions. These findings highlight the need for the development of public health strategies focused on correct diagnosis and early treatment of congenital cardiopathies. leading to a reduction in mortality. (Arq Bras Cardiol. 2020; 115(6):1164-1173)

## Introduction

In the year 2000. 10.65 million of all-cause deaths were recorded in people under the age of 20 worldwide. In 2015. this number declined to 6.65 million. as the mortality rate decreased from 443.76 per 100.000 inhabitants to 269.38 during this period.^1^ This reduction has been attributed to improvements in access to healthcare and education and to a decline in poverty and fertility.^2 - 4^ In addition to this decline in the global mortality rate. there have been changes in the causes of death – while the main cause of deaths was once infectious diseases. perinatal causes. such as prematurity and malformations. have become the most common causes. especially in countries with higher economic development.^2^

Of all deaths due to congenital malformations. malformations of the circulatory system (MCS) are more likely to impact on mortality reduction. given that mortality can be avoided with correct diagnosis and treatment.^6^ In 2015. MCS accounted for 43% of deaths from congenital malformations in people younger than 20 years. Of children who are born with congenital heart disease without receiving medical intervention. 14% do not survive the first month of life and 30% do not survive the first year.^8^

At the same time that mortality in people under 20 years of age has declined in Brazil in recent decades. the relative importance of congenital malformations has increased. rising from the fourth leading cause of death in 2000 to the third in 2015 accounting for 40% of the total of these deaths in the latter year.^9^

Mortality from MCS generally occurs in children under 1 year of age and between ages 1 and 4. given that MCS are often incompatible with life and highly dependent on adequate medical and hospital support.^11^ The knowledge of demographic characteristics of patients who died from MCS would help in the improvement of healthcare and mortality reduction. The objective of this study was to describe the distribution of mortality from MCS by sex. age group. and region in people under age 20 during the period between 2000 and 2015.

## Materials and Methods

This was a descriptive study of mortality rates (number of deaths per 100.000) and proportional mortality (PM) due to MCS. other congenital malformations (OCM). circulatory system disease (CSD). ill-defined causes (IDC). and external causes (EC) in people under age 20 in Brazil. between 2000 and 2015. Information regarding deaths was obtained from the website of the Department of Informatics of the Brazilian Unified Health System (DATASUS) (http://tabnet.datasus.gov.br/cgi/sim/dados/cid10_indice.htmdados).^12^ Data set consisted of the compilation of all death certificates registered in each year between 2000 and 2015. by geoeconomic microregion of Brazil. The cause of death codes used were those defined by the World Health Organization (WHO)’s International Statistical Classification of Diseases and Related Health Problems. 10^th^revision (CID-10).^13^

Information about the populations was obtained from the Brazilian Institute for Geography and Statistics (IBGE) website (https://www.ibge.gov.br/apps/populacao/projecao).^14^ where projections based on censuses are available from the year 2000 to the year 2060 by Brazilian macroregion. sex. age group. and overall totals. Data between 2000 and 2015 were used in the study. considering both sexes. all Brazilian macroregions. and the following age groups: 0-4. 5-9. 10-14. and 15-19 years.^15^ The study period was determined based on the availability of population information. which became consistently available on the IBGE website from the year 2000 on.

Information about deaths was obtained for each geo-economic macroregion. both sexes. and all age groups under 20 (under 1 year of age. 1 to 4 years. 5 to 9 years. 10 to 14 years. and 15 to 19 years) defined by the WHO.^15^ Thus. the mortality rate in the 1-4 age group was calculated using an approximation. subtracting the number of live births by sex and region from the total population age 0-4 years. Mortality rates in children under the age of 1 year are the same as infant mortality. given that the denominator was the number of live births. Information regarding live births by sex and region for the period 2000-2015 was obtained from the DATASUS website.^16^

Deaths caused by diseases of the ICD-10 chapter XVII were divided into MCS and OCM.^13^ Deaths from MCS were classified into the following categories: malformations of cardiac connections (Q20). cardiac septa (Q21). pulmonary and tricuspid valves (Q22). aortic and mitral valves (Q23). others (Q24. except Q24.9 and unspecified Q24.9). great arteries (Q25). and other vessels (Q26-Q28). CSD correspond to ICD-10 chapter IX. IDC correspond to chapter XVIII and EC correspond to ICD-10 chapters XIX and XX.

PM. defined as the ratio between deaths due to a specific cause and deaths due to all causes. was calculated in two ways: total PM (PMt). whose denominators included all causes of death. and endogenous proportional mortality (PMe). whose denominators exclude external causes. Mortality rates (per 100.000) were estimated using the ratio between deaths due to a specific cause and the estimated populations. PMt and mortality rates were estimated by sex. age group. and macroregion for the period 2000-2015. while PMe was obtained on an annual basis. by sex. age group. and macroregion.

The Microsoft Office Excel^17^ and Stata® version 14^18^ were used for calculations.

This study was carried out in accordance with ethical standards and was approved by the Research Ethics Committee of the Clementino Fraga Filho University Hospital. which belongs to the Federal University of Rio de Janeiro.

## Results

In Brazil. from 2000 to 2015. there were 1.367.355 deaths due to all causes in people under age 20. of which 845.481 were male and 521.874 were female. The rate of deaths under 1 year of age fell from 61.41% in 2000 to 51.22% in 2015. The relative frequency of death was higher for males in all age groups. with a frequency 3.8 times higher in the 15-19 group. Mean annual all-cause mortality was 126 per 100.000 inhabitants in both sexes; of these deaths. 61.7% occurred in males.

A total 144.057 deaths from congenital malformation of an organ or system were registered. 85.8% of them in children under 1 year of age. with a similar distribution between the sexes. Of these deaths. 57.892 (39.05) were caused by MCS. Mortality from malformation during the first year of life was 5.7 times higher than all other age groups. with MCS 5.0 times more common and OCM 6.4 times more common. Annual mortality from MCS was 5.3 deaths per 100.000 inhabitants in people under age 20 in both sexes. 5.0 in females and 5.6 in males. PM from MCS was 4.2% in people under age 20 in both sexes. 5.1% in females and 3.7% in males.

In people younger than 20. CSD was the cause of 29.904 deaths in Brazil. 13.198 of which in females and 16.706 in males. PM from CSD was 2.2% in both sexes. 2.5% in females and 2.0% in males. In children under 1 year of age. the risk of death from CSD was 14.7 per 100.000 live births; this number declined in subsequent age groups. reaching 3.9 per 100.000 inhabitants in the 15-19 group. On the other hand. PM increased from 1.4% in children under age 1 to 3.5% in the 15-19 age group.

In Brazil. 85.458 deaths were attributed to IDC. which corresponds to 6.2% of all deaths in people under age 20. Of these deaths. 35.518 occurred in females and 49.940 in males. The risk of death from IDC in both sexes in children under 1 year of age was 95.04 per 100.000 live births; this number declined in subsequent age groups. reaching 5.09 per 100.000 inhabitants in the 15-19 group. On the other hand. PM increased from 6% in children under age 1 to 10.8% in the 1-4 age group. with a progressive decline in the other age groups.

There were 340.974 deaths from EC. 274.627 (80.5%) of which occurred in males and 66.347 (20%) in females. In Brazil. deaths from EC in people under age 20 increased progressively in each age group. in both sexes; this was. however. more pronounced in males. Compared with the under one-year-old group. PM in the 15-19 age group was 31 times greater in males and 18.5 greater in females.

Results of PM and mortality rates by cause of death. age group. sex. and region are shown in Tables 1 [Table t2001] [Table t3001] [Table t4001] [Table t5001] to 6 . In the south and central-west regions of Brazil. the risk of death from MCS was almost twice as high as in the north and northeast regions in children under 1 year of age; the risk declined progressively with age group.

**Table 1:** – Proportional mortality and mortality rates in children according to cause of death. sex. and age group in the north region of Brazil. 2000-2015

Causes of death		Male					Female				
		<1 year	1-4 years	5-9 years	10-14 years	15-19 years	<1 year	1-4 years	5-9 years	10-14 years	15-19 years
MCS	Deaths PM(%) Mort100.000	2,564 *4.9* *100.5*_(1)_	282 *2.7* *2.0*_(2)_	83 *1.5* *0.6*	55 *0.9* *0.4*	51 *0.2* *0.4*	2,064 *5.06* *85.3*_(1)_	286 *3.3* *2.1*_(2)_	64 *1.6* *0.5*	66 *1.6* *0.5*	39 *0.6* *0.3*
Other MC	Deaths PM(%) Mort100.000	4,047 *7.7* *158.7*_(1)_	261 *2.5* *1.8*_(2)_	67 *1.2* *0.5*	50 *0.8* *0.4*	53 *0.2* *0.4*	3,710 *9.1* *153.4*_(1)_	244 *2.8* *1.8*_(2)_	60 *1.5* *0.4*	44 *1.1* *0.3*	26 *0.4* *0.2*
DCS	Deaths PM(%) Mort100.000	496 *0.9* *19.4*_(1)_	217 *2.1* *1.5*_(2)_	180 *3.3* *1.2*	307 *5.0* *2.2*	635 *2.9* *4.8*	426 1.0 17.6_(1)_	233 2.66 1.69_(2)_	159 4.07 1.15	272 *6.54* *2.03*	457 *6.6* *3.6*
Ill-defined	Deaths PM(%) Mort100.000	4,548 *8.6* *17.3*_(1)_	1,845 *17.6* *12.8*_(2)_	683 *12.5* *4.8*	722 *11.8* *5.2*	1,475 *6.6* *11.1*	3,452 *8.5* *142.7*_(1)_	1,514 *17.3* *11.0*_(2)_	499 *12.8* *3.6*	493 *11.9* *3.7*	694 *10.0* *5.4*
External	Deaths PM(%) Mort100.000	837 *1.6* *32.8*_(1)_	2,341 *22.4* *16.3*_(2)_	2,133 *38.9* *14.8*	2,794 *45.6* *20.1*	16,668 *75.0* *125.5*	602 *1.5* *24.9*_(1)_	1,402 *16.0* *10.9*_(2)_	1,180 *30.2* *8.5*	1,373 *33.0* *10.3*	2,517 *36.4* *19.7*
All causes	Deaths PM(%) Mort100.000	52,729 *100.0* *2,067.2*_(1)_	10,459 *100.0* *72.7*_(2)_	5,476 *100.0* *38.1*	6,130 *100.0* *44.1*	22,218 *100.0* *167.3*	40,754 *100.0* *1,684.9*_(1)_	8,750 *100.0* *63.5*_(2)_	3,911 *100.0* *28.4*	4,158 *100.0* *31.1*	6,914 *100.0* *54.0*

MCS: malformations of the circulatory system; Other MC: other congenital malformations excluding MCS; CSD: circulatory system disease; PM(%):proportional mortality;Mort100,000: mortality rate per 100,000 (1) Mortality per 100,000 live births (2) Mortality per 100,000 in the population aged from 0 to 4 years excluding live births

**Table 2 t2001:** – Proportional mortality and mortality rates in children according to cause of death, sex and age group in the northeast region of Brazil, 2000-2015

Causes of death		Male					Female				
		<1 year	1-4 years	5-9 years	10-14 years	15-19 years	<1 year	1-4 years	5-9 years	10-14 years	15-19 years
MCS	Deaths PM(%) Mort100,000	6,743 *4.5* *93.3*_(1)_	822 *3.4* *2.4*_(2)_	255 *1.8* *0.6*	182 *1.0* *0.4*	193 *0.2* *0.4*	5,602 *4.9* *81.5*_(1)_	937 *4.6* *2.8*_(2)_	234 *2.3* *0.6*	180 *1.6* *0.4*	157 *0.8* *0.4*
Other MC	Deaths PM (%) Mort100,000	11,086 *7.5* *153.4*_(1)_	758 *5.7* *2.2*_(2)_	256 *1.9* *0.6*	175 *1.0* *0.4*	171 *0.2* *0.4*	9819 *8.6* *143.0*_(1)_	770 *3.7* *2.3*_(2)_	223 *2.2* *0.5*	172 *1.6* *0.4*	151 *0.8* *0.4*
DCS	Deaths PM(%) Mort100,000	1,138 *0.8* *15.7*_(1)_	721 *3.0* *2.1*_(2)_	565 *4.1* *1.3*	1,005 *5.6* *2.3*	2,374 *3.0* *5.6*	986 *0.9* *14.4*_(1)_	692 *3.4* *2.1*_(2)_	493 *5.0* *1.2*	845 *7.7* *2.0*	1,595 *8.0* *3.8*
Ill-defined	Deaths PM(%) Mort100,000	12,576 *8.5* *174.1*_(1)_	3,403 *14.2* *9.9*_*(* 2)_	1,378 *10.0* *3.2*	1,429 *7.9* *3.3*	3,357 *4.3* *7.9*	9,545 *8.4* *139.0*_*(1)*_	2,931 *14.3* *8.9*_(2)_	1,071 *10.8* *2.6*	1,118 *10.1* *2.7*	1,800 *9.1* *4.3*
External	Deaths PM(%) Mort100,000	1,994 *1.3* *27.6*_(1)_	4,895 *20.4* *14.3*_(2)_	5,245 *38.1* *12.3*	9,119 *50.5* *21.1*	62,854 *80.3* *147.4*	1,372 *1.2* *20.0*_(1)_	3,044 *14.8* *9.2*_(2)_	2,627 *26.4* *6.4*	3,380 *30.7* *8.1*	7,621 *38.3* *18.3*
All causes	Deaths PM(%) Mort100,000	148,346 *100* *2053.1*_(1)_	23,957 *100* *70.0*_(2)_	13,782 *100* *32.0*	18,038 *100* *42.0*	78,248 *100* *183.5*	113,735 *100* *1,656.6*_*(1)*_	20,529 *100* *62.1*_(2)_	9,949 *100* *24.1*	11,019 *100* *26.4*	19,872 *100* *47.8*

*MCS: malformations of the circulatory system; Other MC: other congenital malformations excluding MCS; CSD: circulatory system disease; PM(%):proportional mortality;Mort100,000: mortality rate per 100,000 (1) Mortality per 100,000 live births (2) Mortality per 100,000 in the population aged from 0 to 4 years excluding live births*

**Table 3 t3001:** – Proportional mortality and mortality rates in children according to cause of death, sex, and age group in the southeast region of Brazil, 2000-2015

Causes of death		Male					Female				
		<1 year	1-4 years	5-9 years	10-14 years	15-19 years	<1 year	1-4 years	5-9 years	10-14 years	15-19 years
MCS	Deaths PM(%) Mort100,000	10,534 *7.1* *110.0*_(1)_	1,091 *4.7* *2.7*_(2)_	250 *1.8* *0.5*	250 *1.2* *0.5*	218 *0.2* *0.4*	9,088 *7.8* *99.5*_(1)_	1,101 *5.7* *2.8*_(2)_	275 *2.6* *0.5*	218 *1.7* *0.4*	183 *0.7* *0.3*
Other MC	Deaths PM(%) Mort100,000	15,407 *10.4* *160.8*_(1)_	1,252 *5.4* *3.1*_(2)_	366 *2.6* *0.7*	301 *1.5* *0.6*	290 *0.3* *0.5*	14,042 *12.0* *153.7*_(1)_	1,246 *6.4* *3.2*_(2)_	372 *3.5* *0.7*	288 *2.3* *0.5*	263 *1.1* *0.5*
DCS	Deaths PM(%) Mort100,000	1,537 *1.0* *16.0*_(1)_	818 *3.6* *2.0*_(2)_	533 *3.8* *1.0*	980 *4.8* *1.8*	2,579 *2.6* *4.6*	1,401 *1.2* *15.3*_(1)_	784 *4.1* *2.0*_(2)_	534 *5.0* *1.1*	723 *5.7* *1.4*	1585 *6.5* *2.9*
Ill-defined	Deaths PM(%) Mort100,000	6,054 *4.1* *63.2*_(1)_	1,821 *7.9* *4.5*_(2)_	720 *5.1* *1.4*	1,166 *5.7* *2.2*	3,856 *3.9* *6.9*	4,441 *3.8* *48.6*_(1)_	1,406 *7.3* *3.6*_(2)_	650 *6.1* *1.3*	837 *6.* *6* *1.6*	1,610 *6.6* *3.0*
External	Deaths PM(%) Mort100,000	4,969 *3.4* *51.9*_(1)_	5,237 *22.8* *12.8*_(2)_	5,166 *36.5* *9.8*	10,487 *51.0* *19.4*	80,167 *80.4* *143.6*	3,237 *2.8* *35.4*_(1)_	3,470 *17.9* *8.9*_(2)_	2,897 *27.3* *5.7*	4,158 *32.9* *8.0*	10,563 *43.0* *19.5*
All causes	Deaths PM(%) Mort100,000	147,871 *100* *1,543.8*_(1)_	23,006 *100* *56.3*_(2)_	14,167 *100* *26.9*	20,571 *100* *38.0*	99,661 *100* *178.6*	116,679 *100* *1,277.4*_(1)_	19,333 *100* *49.4*_(2)_	10,663 *100* *2.1*	12,650 *100* *24.2*	24,550 *100* *45.3*

*MCS: malformations of the circulatory system; Other MC: other congenital malformations excluding MCS; CSD: circulatory system disease; PM(%):proportional mortality;Mort100,000: mortality rates per 100,000 (1) Mortality per 100,000 live births (2) Mortality per 100,000 in the population aged from 0 to 4 years excluding live births*

**Table 4 t4001:** – Proportional mortality and mortality rates in children according to cause of death, sex, and age group in the south region of Brazil, 2000-2015

Causes of death		Male					Female				
		<1 year	1-4 years	5-9 years	10-14 years	15-19 years	<1 year	1-4 years	5-9 years	10-14 years	15-19 years
MCS	Deaths PM(%) Mort100,000	3,869 *8.5* *120.8*_(1)_	429 *5.5* *3.1*_(2)_	114 *2.2* *0.6*	94 *1.2* *0.5*	127 *0.4* *0.6*	3,147 *8.7* *103.1*_(1)_	409 *6.6* *3.1*_(2)_	114 *3.1* *0.7*	80 *1.7* *0.4*	76 *0.9* *0.4*
Other MC	Deaths PM(%) Mort100,000	5,694 *12.5* *177.8*_(1)_	590 *7.6* *4.3*_(2)_	188 *3.7* *1.0*	147 *2.0* *0.8*	141 *0.4* *0.7*	5,093 *14.1* *167.0*_(1)_	495 *8,0* *3,8*_(2)_	158 *4.3* *0.9*	145 *3.1* *0.8*	116 *1.3* *0.6*
DCS	Deaths PM(%) Mort100,000	257 *0.6* *8.0*_(1)_	191 *2.5* *1.4*_(2)_	123 *2.4* *0.7*	255 *3.4* *1.3*	618 *2.0* *3.2*	233 *0.6* *7.6*_(1)_	184 *2,9* *1,4*_(2)_	118 *3.2* *0.7*	197 *4.3* *1.1*	358 *4.1* *1.9*
Ill-defined	Deaths PM(%) Mort100,000	1,802 *4.0* *56.3*_(1)_	375 *4.8* *2.7*_(2)_	130 *2.5* *0.7*	210 *2.8* *1.1*	577 *1.8* *3.0*	1,388 *3.9* *45.5*_(1)_	370 *5.9* *2.8*_(2)_	110 *3.0* *0.6*	163 *3.5* *0.9*	293 *3.3* *1.6*
External	Deaths PM(%) Mort100,000	2,037 *4.5* *63.6*_(1)_	2,211 *28.5* *16.0*_(2)_	2,265 *44.3* *12.5*	4,272 *56.8* *22.5*	25,974 *82.3* *133.1*	1,532 *4.3* *50.2*_(1)_	1,332 *21.4* *10.1*_(2)_	1,250 *33.9* *7.2*	1,825 *39.5* *10.0*	4,398 *50.3* *23.4*
All causes	Deaths PM(%) Mort100,000	45,436 *100* *1,419.1*_(1)_	7,749 *100* *56.3*_(2)_	5,113 *100* *28.2*	7,514 *100* *39.6*	31,541 *100* *161.6*	36,020 *100* *1,180.9*_(1)_	6,228 *100* *47.3*_(2)_	3,692 *100* *11.2*	4,621 *100* *25.4*	8,738 *100* *46.5*

*MCS: malformations of the circulatory system; Other MC: other congenital malformations excluding MCS; CSD: circulatory system disease; PM(%):proportional mortality;Mort100,000: mortality rates per 100,000 (1) Mortality per 100,000 live births (2) Mortality per 100,000 in the population from 0 to 4 years excluding live births*

**Table 5 t5001:** – Proportional mortality and mortality rates in children according to cause of death, sex, and age group in the central-west region of Brazil, 2000-2015

Causes of death		Male					Female				
		<1 year	1-4 years	5-9 years	10-14 years	15-19 years	<1 year	1-4 years	5-9 years	10-14 years	15-19 years
MCS	Deaths PM(%) Mort100,000	2,434 *8.0* *129.8*_(1)_	277 *4.9* *3.5*_(2)_	64 *1.9* *0.6*	44 *1.0* *0.4*	52 *0.3* *0.5*	2,115 *8.8* *118.6*_(1)_	239 *5.2* *3.2*_(2)_	41 *1.8* *0.4*	49 *1.7* *0.5*	51 *1.0* *0.5*
Other MC	Deaths PM(%) Mort100,000	3,481 *11.4* *185.7*_(1)_	272 *4.8* *3.5*_(2)_	79 *2.3* *0.8*	52 *1.1* *0.5*	53 *0.3* *0.5*	3,065 *12.7* *171.9*_(1)_	254 *5.5* *3.4*_(2)_	57 *2.5* *0.6*	71 *2.5* *0.7*	44 *0.8* *0.4*
DCS	Deaths PM(%) Mort100,000	307 *1.0* *16.4*_(1)_	137 *2.4* *1.7*_(2)_	92 *2.7* *0.9*	202 *4.5* *2.0*	439 *2.3* *4.2*	234 *1.0* *13.1*_(1)_	152 *3.3* *2.0*_(2)_	72 *3.1* *0.7*	160 *5.6* *1.6*	305 *5.8* *3.0*
Ill-defined	Deaths PM(%) Mort100,000	927 *3.0* *49.4*_(1)_	248 *4.4* *3.2*_(2)_	108 *3.2* *1.1*	152 *3.4* *1.5*	378 *1.9* *3.7*	588 *2.4* *33.0*_(1)_	210 *4.6* *2.8*_(2)_	78 *3.4* *0.8*	89 *3.1* *0.9*	168 *3.2* *1.7*
External	Deaths PM(%) Mort100,000	979 *3.2* *52.* *2*_(1)_	1,620 *28.5* *20.7*_(2)_	1,575 *46.8* *15.8*	2,615 *57.8* *25.9*	16,173 *83.6* *156.5*	688 *2.9* *38.6*_(1)_	1,080 *23.5* *14.4*_(2)_	847 *36.8* *8.9*	1,256 *44.1* *12.9*	2,696 51.0 26.7
All causes	Deaths PM(%) Mort100,000	30,511 *100.0* *1,627.5*_(1)_	5,683 *100.0* *72.5*_(2)_	3,366 *100.0* *33.8*	4,522 *100.0* *44.8*	19,348 *100.0* *187.2*	24,081 *100.0* *1,350.7*_(1)_	4,590 *100.0* *61.2*_(2)_	2,303 *100.0* *24.1*	2,845 *100.0* *29.2*	5,290 *100.0* *52.4*

*MCS: malformations of the circulatory system; Other MC: other congenital malformations excluding MCS; CSD: circulatory system disease; PM(%):proportional mortality; Mort100,000: mortality rates per 100,000 (1) Mortality per 100,000 live births (2) Mortality per 100,000 in the population aged from 0 to 4 years excluding live births*

**Table 6:** – Proportional mortality and mortality rates in children according to cause of death, sex, and age group, Brazil, 2000-2015

Causes of death		<20 years	Male						Female					
		Total	Total	<1 year	1-4 years	5-9 years	10-14 years	15-19 years	Total	<1 year	1-4 years	5-9 years	10-14 years	15-19 years
MCS	Deaths PM(%) Mort100,000	57,892 *4.2* *5.3*	31,077 *3.7* *5.6*	26,144 *6.2* *107.0*_(1)_	2,901 *4.1* *2.7*_(2)_	766 *1.8* *0.6*	625 *1.1* *0.4*	641 *0.3* *0.5*	26,815 *5.1* *5.0*	22,016 *6.6* *94.7*_(1)_	2,972 *5.0* *2.9*_(2)_	728 *2.4* *0.5*	593 *1.7* *0.4*	506 *0.8* *0.4*
Other MC	Deaths PM(%) Mort100,000	86,165 *6.3* *7.9*	45,237 *5.4* *8.2*	39,715 *9.3* *162.6*_(1)_	3,133 *4.4* *2.9*_*(* 2)_	956 *2.3* *0.7*	725 *1.3* *0.5*	708 *0.3* *0.5*	40,928 *7.78* *7.7*	35,729 *10.8* *153.7*_(1)_	3,009 *5.1* *2.9*_(2)_	870 *2.9* *0.7*	720 *2.0* *0.5*	600 *0.9* *0.4*
DCS	Deaths PM(%) Mort100,000	29,904 *2.2* *2.8*	16,706 *2.0* *3.0*	3,735 *0.9* *15.3*_(1)_	2,084 *2.9* *1.9*_(2)_	1,493 *3.6* *1.1*	2,749 *4.8* *2.0*	6,645 *2.6* *4.7*	13,198 *2.54* *2.5*	3,280 *1.0* *14.1*_(1)_	2,045 *3.4* *2.0*_(2)_	1,376 *4.5* *1.0*	2,197 *6.2* *1.6*	4,300 *6.6* *3.1*
Ill-defined	Deaths PM(%) Mort100,000	85,458 *6.2* *7.9*	49,940 *5.9* *9.0*	25,907 *6.1* *106.0*_(1)_	7,692 *10.9* *7.0*_(2)_	3,019 *7.2* *2.2*	3,679 *6.5* *2.6*	9,643 *3.8* *6.8*	35,518 *6.82* *6.7*	19,414 *5.9* *83.5*_(1)_	6,431 *10.8* *6.2*_(2)_	2,408 *7.9* *1.8*	2,700 *7.7* *2.0*	4,565 *7.0* *3.3*
External	Deaths PM(%) Mort100,000	340,974 *24.9* *31.4*	274,627 *32.5* *49.7*	10,816 *2.5* *44.3*_(1)_	*16,304 23.0 15.0*_(2)_	16,384 *39.1* *11.9*	29,287 51.6 20.9	201,836 *80.4* *142.5*	66,347 *12.75* *12.5*	7,431 *2.2* *32.0*_(1)_	10,328 *17.4* *9.9*_(2)_	8,801 *28.8* *6.6*	11,992 *34.0* *8.9*	27,795 *42.5* *20.2*
All causes	Deaths PM(%) Mort100,000	1,367,355 *100.0* *126.0*	845,481 *100.0* *153.0*	424,932 *100.0* *1,739.3*_(1)_	70,854 *100.0* *65.2*_(2)_	41,904 *100.0* *30.4*	56,775 *100.0* *40.5*	251,016 100.0 177.3	521,874 *100.0* *98.0*	331,269 *100.0* *1,424.7*_(1)_	59,430 *100.0* *57.0*_(2)_	30,518 *100.0* *23.0*	35,293 *100.0* *26.1*	65,364 *100.0* *47.6*

*MCS: malformations of the circulatory system; OtherMC: other congenital malformations excluding MCS; CSD: circulatory system disease; PM(%):proportional mortality;Mort100,000:*

PMe from MCS (which did not include EC) increased 1.5-fold in children under 1 year of age in both sexes in the south. southeast. and central-west regions from 2000 to 2015. This number increased 2.6-fold in the north region and 3.2-fold in the northeast region. Results are described regardless of sex since no differences were found between men and women. In other age groups. variations over time were small. with a few isolated peaks. due to low death frequencies. In children under 1 year of age. there was a 4.6% difference in PMe between the south and the northeast regions in the year 2000; this number declined to 2.8% in 2015. Similarly. in the 15-19 group. PMe between the south and the northeast regions fell from 1.7% in 2000 to 0.6% in 2015 ( Figure 1 ). In general. the differences in PMe between regions decreased in all age groups. especially in the last years observed.

Regarding MCS. in all regions and regardless of sex and age. the highest PM occurred without a precise diagnosis. denominated unspecified according to the ICD-10. In the north and northeast regions. more than 60% of deaths from MCS were not specifically classified (Q24.9). The second most frequent category of MCS was malformation of cardiac septa. in all geographic regions. independent of sex and age; this was most pronounced in the southeast region. with a frequency of 13% ( Figure 2 ).

**Figure 2:** – Proportional mortality from malformations of the circulatory system in individuals under 20 years. by sex and macroregion of Brazil. 2000 to 2015

## Discussion

In Brazil. between 2000 and 2015. over half (55%) of deaths in people under 20 were concentrated in children under 1 year of age. This demonstrates the extent to which this age group is vulnerable. We observed a similar distribution pattern of mortality from MCS with respect to age group and sex in different regions of Brazil. The same applies to other causes of death. However. the relative importance of large groups of causes of death varied in different manners with age. regardless of sex. In people under 20. malformations. MCS. and OMC decreased in importance as age increased. In contrast. mortality from CSD showed an opposite pattern. EC showed a “J” curve and IDC showed little variation. peaking in the 1-4 age group.

Even though the risk of all-cause mortality was higher in males. the relative importance of death from malformations and CSD was higher in females on account of the higher prevalence of EC among males. which increased with age. This observation is in agreement with previous studies;^19^ relatively high mortality from EC in men has been reported in different locations around the world.^1^ This may be attributed to men’s greater exposure to risk factors. such as accidents. alcohol consumption. use of tobacco and other drugs. use of firearms and other weapons. truancy and dropping out. and involvement illicit activities.^21^

The distribution of PM from MCS throughout the regions of Brazil was in accordance with that reported in Latin America. According to the Global Burden of Disease (GBD) study. in 2015. PM from MCS in people under age 20 was 9.7% in Mexico. followed by the Southern Cone (Argentina. Chile. and Uruguay) with 7.8%. Brazil with 6.5%. the Andean Region comprising Bolivia. Ecuador. and Peru with 5.8%. and the Caribbean with 4.4%.^1^ Thus. regions with higher indexes of poverty have higher percentages of death from MCS. which may be attributed to a lower diagnostic capacity. given that diagnosis of MCS requires adequate medical and hospital support.^5^ There is a noticeable difference between the percentage shown in this study and that of the GBD. because the latter compiled complete data from only eight states in Brazil and estimated data for the rest.^3^

Given the progress of PMe over time. it seems that a correction of the low percentage of MCS diagnoses in death occurred in all regions. mainly in the north and northeast regions. especially in children under age 1. when mortality from MCS most commonly occurs. However. the percentages of imprecise anatomical and functional diagnoses of MCS. classified as unspecified. continue to be greater in these regions. In addition. the highest percentages of deaths from IDC were also in the north and northeast regions in children under age 5. Thus. it is necessary to improve diagnostic methods. especially in the poorest regions of the country.

Of all causes of death from MCS. unspecified causes were the most frequent in both sexes and in all age groups and regions. which suggests low levels of access to prenatal and newborn diagnoses. According to the Brazilian Society of Pediatrics. 1-2 of every 1.000 live births have critical congenital heart diseases. but 30% of these cases are discharged from the hospital without a correct diagnosis. and may evolve into shock. hypoxia. or premature death before adequate treatment is provided.^22^ Prenatal care and obstetric echocardiography could reduce these deaths by making early diagnosis and referral to specialized treatment centers possible. even before birth.^23^

The decline in some regional differences. indicated by the PMe in the last years of the study period. may be attributed to public health measures for the detection of congenital heart diseases. such as pulse oximetry^23^ in newborns with gestational age above 34 weeks. which has been recommended since 2011. and was incorporated into the Brazilian Unified Health System’s list of procedures in 2014.^24^ Another exam is fetal echocardiography routinely performed in pregnant women aged over 35 or with other risk factors for fetal malformation.^25^ In 2004. a “Pact for the Reduction of Maternal and Neonatal Mortality” was signed on the three levels of government in Brazil with the goal of reducing neonatal mortality. The strategies were designed to reduce mortality. with greater emphasis placed on the north and northeast regions.^26^

There was an inverse trend of CSD with MCS. and the importance of CSD increased as age increased. It is worth mentioning that children with MCS who survive the first year of life. even with treated for MCS. may develop complications and sequelae. such as heart failure. arrhythmia. endocarditis. and other CSD. which may lead to death during adolescence. and increase mortality from CSD.^27^ The lowest differences in the MCS:CSD ratios were found are in the northeast region. followed by the north region. This may be explained by misdiagnoses of MCS and CSD. since a correct differential diagnosis relies on appropriate. timely diagnostic resources. which are scarcer in these two regions.

One limitation of this study was the age-group division provided by the IBGE. based on population projections. that was adopted in the study. In this categorization. children under 1 year of age was included in the same age group of children under 5 years of age. and hence values of children from ages 1 to 4 were approximated to values slightly lower than the real ones. Thus. the risks measured by the mortality rates per 100.000 in this age group were slightly overestimated. However. this fact did not affect the estimates of PMs. as they do not depend on population estimates. Another limitation is the quality of information on the causes of death provided in death certificates. Data on the time between diagnosis and death were incomplete. and for this reason. it was not possible to determine the influence of the inaccurate diagnosis of MCS on death. Death certificates are. however. the only comprehensive source of data regarding deaths in Brazil as a whole.

## Conclusion

In Brazil. from 2000 to 2015. in people under age 20. MCS were the leading cause of death among all malformations. being twice as important as CSD. especially in children under 1 year of age. There were improvements in diagnoses of death from MCS in the final years of the study period. However. in MCS. the frequency of imprecise diagnoses is still elevated in both sexes. in all age groups. and especially in the north and northeast regions of Brazil. Thus. public health strategies must be strengthened. and that greater attention must be given to newborns aiming the correct diagnosis and early treatment of congenital cardiopathies.

**Figura 1:** – Endogenous proportional mortality due to malformations of the circulatory system by age group and macroregion of Brazil. 2000 to 2015; *PMe%= endogenous proportional mortality due to malformations of the circulatory system. excluding external causes
